# Peripheral T follicular helper Cells Make a Difference in HIV Reservoir Size between Elite Controllers and Patients on Successful cART

**DOI:** 10.1038/s41598-017-17057-y

**Published:** 2017-12-01

**Authors:** Marcial García, Miguel Górgolas, Alfonso Cabello, Vicente Estrada, José Manuel Ligos, Manuel Fernández-Guerrero, Carlos Barros, Juan Carlos López-Bernaldo, Francisco Javier De La Hera, María Montoya, José Miguel Benito, Norma Rallón

**Affiliations:** 1 Instituto de Investigación Sanitaria Fundación Jiménez Díaz, Universidad Autónoma de Madrid (IIS-FJD, UAM), 28040 Madrid, Spain; 2 Hospital Universitario Rey Juan Carlos, 28933 Móstoles, Spain; 3 Hospital Universitario Fundación Jiménez Díaz, 28040 Madrid, Spain; 4 Hospital Universitario Clínico San Carlos, 28040 Madrid, Spain; 5 Celomics Unit, Centro Nacional de Investigaciones Cardiovasculares, 28029 Madrid, Spain; 6 Hospital Universitario de Móstoles, 28935 Móstoles, Spain; 7 Hospital General Universitario Gregorio Marañón, 28007 Madrid, Spain

## Abstract

HIV latency is the main barrier to HIV eradication. Peripheral T follicular helper (pTfh) cells have a prominent role in HIV persistence. Herein, we analyzed the HIV reservoir size within memory CD4+ T-cell subsets in patients with HIV replication control. Twenty HIV-infected patients with suppressed HIV replication were included, with 10 elite controllers (EC) and 10 treated (TX) individuals. The HIV reservoir size was analyzed in resting memory CD4+ T-cells (Trm), pTfh, and non-pTfh cells using an ultrasensitive digital-droplet-PCR assay. Inter-group and intra-group differences were tested using non-parametric tests. Compared with the TX patients, the EC patients had smaller HIV reservoir not only in Trm but also in pTfh and non-pTfh subsets of memory CD4+ T-cells. The largest differences were observed in pTfh cells (p = 0.025). The pTfh and non-pTfh cells harbored similar levels of HIV-DNA in the EC (p = 0.60) and TX patients (p = 0.17); however, the contribution to HIV-DNA levels in memory CD4+ T-cells varied among the pTfh and non-pTfh subsets in both groups of patients. The EC patients showed smaller HIV reservoir in memory CD4+ cells, especially in the pTfh subset, a population of cells with a pivotal role in the antiviral immune response, suggesting a potential link between low levels of infection in pTfh cells and the ability of the EC patients to spontaneously control HIV replication.

## Introduction

The HIV latent reservoir is the main barrier to HIV eradication. Although combination antiretroviral therapy (cART) can effectively block viral replication in the host and reduce the circulating virus to undetectable levels^1^, antiviral therapy cannot completely eliminate the virus from the human body. As a result, the viral load rapidly rebounds within 2 to 8 weeks after cART discontinuation^2–4^. The rebound occurs due to the presence of anatomic reservoirs that drugs cannot easily access^5^. Additionally, there are reservoirs containing cells with HIV integrated into the cell genome. However, the viral genome is transcriptionally silent, and the virus is refractory to both cART and the immune system^6–8^.

The cellular reservoir may include several cell subpopulations, and the latent and long-term persistent virus has been described in resting memory CD4+ T-cells (Trm cells)^9^. This CD4+ T-cell subset with memory phenotype (CD45RO+) and low expression of activation markers such as HLA-DR, CD25 or CD69^9^ allow the establishment of latent reservoir extremely stable with a mean half-life of around 44 months^10,11^. There is another memory CD4+ T-cell subpopulation present in germinal centers, T follicular helper (Tfh) cells. Tfh cells have a prominent role in regulating the HIV reservoir by supporting persistent infection, replication, and production of HIV in both viremic HIV-infected subjects^12^ and long-term cART-treated aviremic individuals^13^. Tfh cells are a memory CD4+ T-cell subset expressing CXCR5 and have B cell helper function localized within secondary lymphoid organs^14^. The counterpart in peripheral blood is called the peripheral Tfh (pTfh) cells because they display functional properties similar to Tfh cells and represent approximately 20% of total memory CD4 T-cells^15–17^. Although several markers have been associated to pTfh cells (CXCR5, CXC3, CCR7, PD1)^15,17–23^, only the expression of CXC chemokine receptor 5 (CXCR5) has been considered as a specific marker for total pTfh cells^15^. Some differences between pTfh and Tfh cells regarding expression of surface markers have been noticed, such as the low expression of PD-1 in pTfh cells^17^ in contrast to the high expression of this marker by Tfh cells^24–26^, suggesting that pTfh cells would be in a resting state similar to Trm cells^17^.

Accessing the lymph nodes of HIV-infected patients is a complex and invasive procedure. Thus, the availability of pTfh cells in blood represents an important step for the understanding of HIV infection and persistence in this compartment. Several studies have focused on the role of pTfh cells in HIV infection^18–20^. However, only one recent study has focused in the role of pTfh cells in HIV persistence in patients before and after the initiation of cART^22^.

Elite controller (EC) patients are a model for the development of therapeutic strategies aimed at functional HIV cure^27^. There is extensive literature on the ability of these subjects to spontaneously maintain HIV replication control^27,28^. A few recent studies evaluated their ability to maintain better control of the HIV reservoir size^29–31^. However, these studies mostly used peripheral blood mononuclear cells (PBMCs)^29,30^ and resting CD4+ T-cell subsets^31^. There are no studies examining pTfh cells, which have a prominent role in HIV reservoir. Therefore, we have characterized the reservoir size in different memory CD4+ T-cell subpopulations. We examined the Trm cells, pTfh cells, and non-pTfh cells from EC patients and compared the results with those from treatment-controlled HIV-patients (TX, aviremic patients with cART).

## Results

### Baseline characteristics of the study population

The EC and TX patient groups were comparable with respect to age, gender, CD4 counts, and length of infection (Table 1). The median follow-up of EC patients maintaining EC status was 7 [3–12] years, and 7 of 10 subjects had more than 5 years of EC status. During the follow-up period, all EC patients showed high and stable CD4 counts (897 [771–1,187] at the beginning and 912 [630–1,438] cells/µL at the end of follow-up, p = 0.86).

**Table 1:** Study group characteristics.

Characteristic	Study Groups		p-value
	EC	TX	
n	10	10	
Age (Years)	46 [40–54]	48 [45–52]	0.43
Male (%)	50	90	0.07
Viral load (copies/mL)	50	50	NA
CD4 counts (cells/μL)	1.235 [629–1474]	820 [626–1.253]	0.63
Length of infection (Years)	13 [10–16]	14 [12–17]	0.66
Length of treatment (Years)	NA	13 [10–17]	—

Data are expressed as Median [IQR], except sex, expressed as %; p-value: comparison between EC and TX group (U-Mann-Whitney test); NA: not apply.

### EC patients have a smaller HIV reservoir than do TX patients

**Figure 1:**
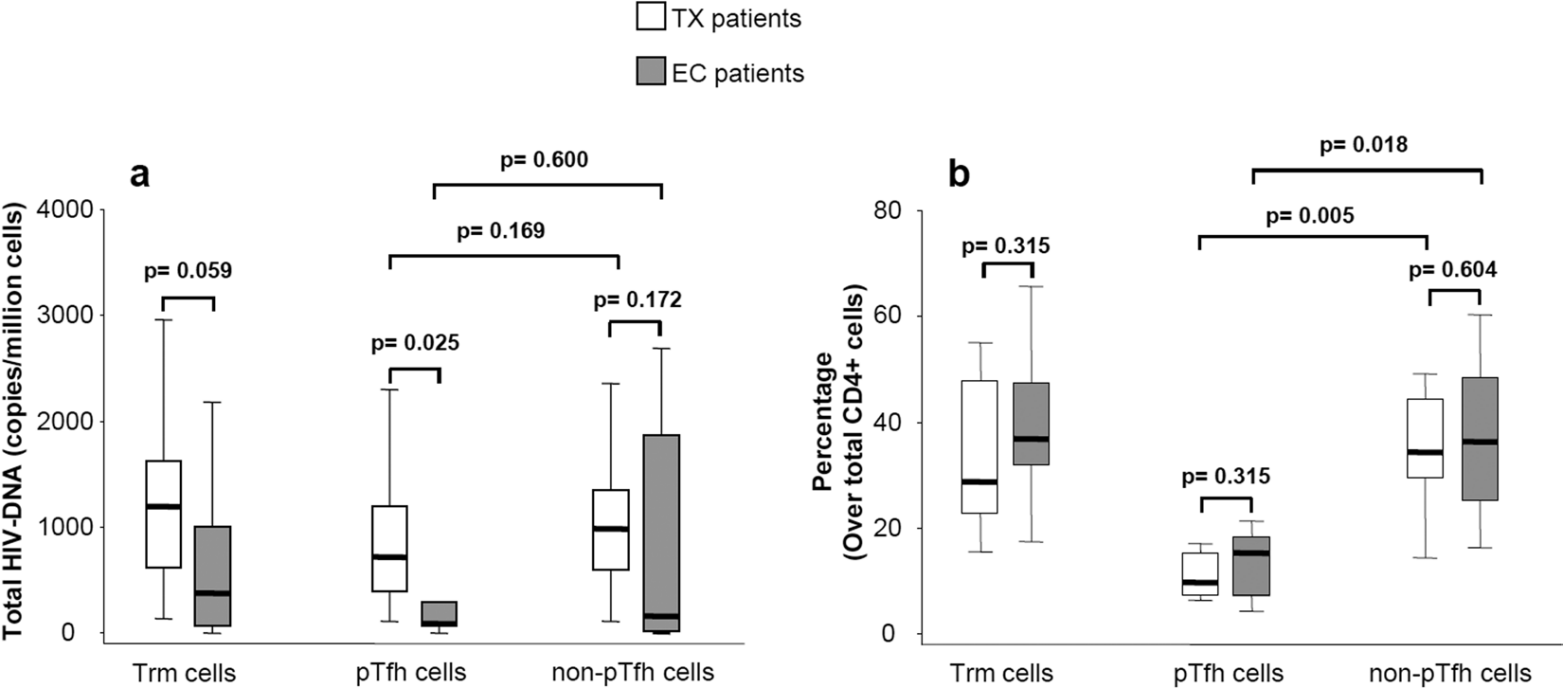
Box-plot graphs showing: (**a**) HIV reservoir size (HIV-DNA copies per million of cells) in Trm, pTfh and non-pTfh cells of the HIV-infected groups with suppressed HIV replication (EC and TX patients). (**b**) Percentage of Trm, pTfh and non-pTfh cells over the total CD4+ T-cells in HIV-infected groups with suppressed HIV replication (EC and TX patients). P-values of comparisons between the EC and TX groups of patients by Mann-Whitney U-test, and between pTfh and non-pTfh cell subsets by Wilcoxon signed- rank test are shown.

We found lower levels of HIV-DNA in Trm cells in the EC group than in those in the TX group (median [IQR]: 381 [74–1,002] vs. 1197 [619–1,623] copies/million cells respectively; p = 0.059), showing that EC maintain a lower HIV reservoir in this cell population than do patients with cART-mediated long-term suppression of HIV replication (Fig. 1a). Furthermore, we found the HIV reservoir size in pTfh cells was significantly lower in patients able to spontaneously control HIV replication (88 [33–291] copies/million cells) than that in patients with cART-mediated viral suppression (723 [393–1,199] copies/million cells; p = 0.025) (Fig. 1a). Interestingly, this difference between EC and TX patients was less pronounced in non-pTfh cells (162 [18–1,871] vs. 989 [600–1,354]) and was not significant (p = 0.172) (Fig. 1a).

### HIV reservoir size is not influenced by frequency of the different analyzed CD4+ T-cell subsets

We evaluated the possibility that HIV reservoir size in EC or TX patients may be influenced by the relative frequency of specific CD4+ T-cell populations. Therefore, we assessed the frequencies of each T-cell subset involved in maintaining the HIV reservoir (Trm, pTfh, and non-pTfh) for both groups of studied patients. The frequencies of Trm, pTfh, and non-pTfh cells were similar for both groups of studied patients (Fig. 1b). We next evaluated the correlation between the size of HIV reservoir in the different CD4+ T-cell subsets and their frequency in EC and TX subjects. There was no correlation between HIV-DNA levels in Trm and the frequency of Trm within the total CD4+ T-cells (Spearman’s rho = 0.02; p = 0.93). Additionally, there was no relationship between HIV-DNA levels in pTfh and the frequency of pTfh in the total CD4+-T-cell population (Spearman’s rho = −0.15; p = 0.56). Similar results were observed in non-pTfh cells (Spearman’s rho = 0.27; p = 0.30).

### pTfh and non-pTfh cell subsets contribute to HIV reservoir size

**Figure 2:**
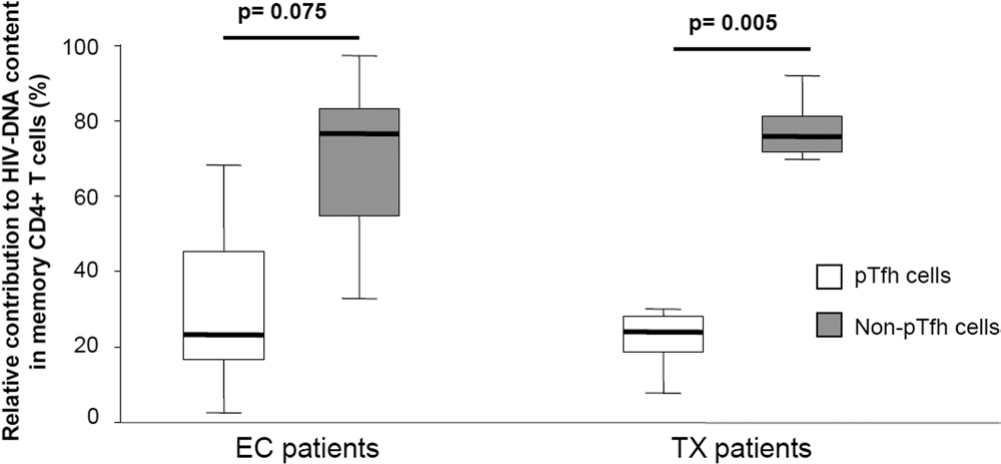
Box-plots graphs showing the relative contribution (percentage) of pTfh and non-pTfh cells to HIV-DNA content in memory CD4+ T-cells in the EC and TX groups of patients. P-values of comparisons between pTfh and non-pTfh by Wilcoxon signed-rank test are shown.

We confirmed the contribution of the Trm cell subset to HIV reservoir size. The Trm cells represent a specific cell subset among memory CD4+ T-cells. We found no statistically significant differences in levels of HIV-DNA (copies/million cells) between pTfh and non-pTfh cells in both EC (pTfh: 88 [33–291] vs non-pTfh: 162 [18–1,871]; p = 0.600) and TX (pTfh: 723 [393–1,199] vs non-pTfh: 989 [600–1,354]; p = 0.169) patients (Fig. 1a). This finding suggests both subsets contribute to HIV persistence. However, among total memory CD4+ T-cells (comprising pTfh and non-pTfh cell subsets) the proportion of non-pTfh was higher than the proportion of pTfh cells in both EC patients (70% [66–78] vs 30% [22–35] respectively; p = 0.018) and TX patients (76% [68–78] vs 24% [22–32] respectively; p = 0.005) (Fig. 1b). Therefore, the relative contribution to the HIV reservoir size in total memory CD4+ T-cells was higher for the non-pTfh cells than the pTfh cells in both EC and TX patients (Fig. 2).

## Discussion

To our knowledge, this is the first study analyzing the levels and relative contribution of HIV-DNA from Trm, pTfh, and non-pTfh subsets of memory CD4+ T-cells to the HIV reservoir size in both patients with long-term spontaneous control of HIV replication (EC) and in patients with cART-mediated long-term suppression of HIV replication (TX). In this study, compared with TX patients, EC patients showed a smaller HIV reservoir. This finding is in agreement with a previous report analyzing levels of HIV-DNA in resting central memory CD4+ T-cells from elite controller patients^31^.

Tfh cells and their counterpart in peripheral blood (pTfh cells) have recently become a key aspect of HIV infection^18–21,32,33^ and the persistent reservoir^12,13,22^. Compared with other subsets of memory CD4+ cells, the Tfh cells located in the lymph nodes of progressing HIV-infected patients harbored higher levels of replication-competent HIV-DNA^12,13^. A recent study found similar results using pTfh cells from progressor patients before and after cART^22^. Interestingly, we found that the HIV reservoir size in pTfh was smaller in patients able to spontaneously control HIV replication than that in patients with cART-mediated viral suppression. This result suggests that host-mediated containment of HIV replication observed in EC patients might reduce the HIV reservoir, especially in the pTfh cell population compared with other memory CD4+ T-cell populations such as non-pTfh cells. Moreover, pTfh cells can support the HIV-specific cellular response^16^ and HIV-specific broadly neutralizing antibody production in HIV-infected typical progressing patients^17^ and in EC subjects^23^. Thus, the low level of HIV infection in pTfh cells from EC patients could help to preserve their functionality and impacts the ability of EC patients to maintain control of HIV replication.

It is also important to note that the study by Perreau *et al*. found that Tfh cells are expanded in HIV-infected subjects and decreased after cART^12^. Therefore, the lower total HIV-DNA that we observed in pTfh of EC patients could be due to a “dilution effect” as a consequence of a higher frequency of pTfh cells in EC. However, our results show that was not the case since frequency of pTfh cells was similar for both EC and TX patients, and there was no correlation between reservoir size in the pTfh cell subset and their relative frequency within CD4+ T-cells.

Moreover, HIV infection is controlled early in EC patients^34,35^. This finding may provide insight into the mechanism explaining the lower HIV reservoir size observed in EC patients compared with that in TX patients in whom cART-induced HIV replication control occurs later since they usually start therapy at least one year after infection. Moreover, since pTfh cells are more supportive for HIV replication^22^ the lower levels of HIV-DNA that we also found in the Trm cells of EC patients could be the consequence of the lower amount of pTfh cells harboring HIV-DNA observed in these patients.

There are several controversial issues in our study. First, the definition of the pTfh cell subset is variable in the literature and this is problematic because it is difficult to compare and interpret results from different studies. Several markers (CXCR5, CXC3, CCR7, PD1) have been associated with pTfh cells^15,17–23^. However, the majority of the markers are not pTfh cell-specific and CXCR3, CCR7 or PD1 have been used in studies to distinguish different pTfh functional states and have found controversial results^17,22,23^. Currently, the most definitive/unambiguous marker available to identify both Tfh^36,37^ and circulating Tfh (pTfh)^15,18,21,22,38^ cells is the expression of CXC chemokine receptor 5 (CXCR5), which includes the distinct pTfh subsets with different capacities to regulate B cell responses. Second, a previous study suggests that EC patients have an excess of unintegrated HIV DNA^30^; thus, the measurement of total HIV-DNA and the absence of virus growth assays to evaluate replication-competent virus in our study could interfere with our data analysis; however, several authors recently noted the relevance of measuring total HIV-DNA as a marker of viral reservoir dynamics with clinical implications^39,40^. The authors suggested that both integrated and total HIV-DNA provides an inducible and functional reservoir and predicts *ex vivo* viral outgrowth^40,41^. Additionally, it has been highlighted that the establishment of HIV latency and virus production from unintegrated genomes follows direct infection of resting CD4+ T-cells^42^. Third, given the relevance of Tfh and pTfh cells in HIV reservoir maintenance^12,13,22^, our finding regarding no appreciable intragroup difference between pTfh cells and non-pTfh cells in HIV-DNA content could sounds controversial. However, since we could not measure replication-competent provirus we cannot rule out the possibility that pTfh may represent a major reservoir as compared to non-pTfh cells in HAART-treated patients, as it has been suggested by Pallikuth *et al*.^22^, and even in EC patients. Finally, a recent study reported that CD32a (the type II receptor for Fc fragment of IgG) is a cell surface marker specific for resting CD4+ T-cells carrying replication-competent HIV^43^. Thus, it will be essential to analyze the expression levels of this marker on the different CD4+ T-cell subsets contributing to HIV reservoir in future studies. It will also be valuable to determine the relationship between CD32a expression and HIV-DNA content in these cell subsets.

In summary, our study results show that elite controller patients maintain low levels of HIV-DNA in the pTfh cell subset, which is a population of memory CD4+ T-cells with special relevance in the maintenance of HIV persistence and in the generation of a robust virus-specific immune response. Our results also highlight the importance of monitoring HIV-DNA in different subsets of memory CD4+ T-cells, given their differential relevance in the phenomenon of viral persistence and their differential contribution to the extent of the HIV reservoir in peripheral blood. These findings may also have clinical relevance in future clinical trials aimed at purging the HIV reservoir, and studies with a larger cohort of patients could support our results.

## Methods

### Study population

This cross-sectional study was conducted in the following groups of chronically HIV-infected patients with suppressed HIV replication: 10 EC with undetectable plasma HIV-RNA (<50 copies/mL) without cART and 10 TX with at least five years of cART and complete viral suppression. The groups were matched for age, sex, length of infection and CD4+ T-cell counts. An HIV-infected patient was considered an EC when having at least three consecutive plasma HIV viral load determinations with no more than 50 HIV-RNA copies/mL during at least 12 months of follow-up in absence of any combination of antiretroviral therapy (cART). The EC patients included in our study had long-term control of HIV replication and stable CD4 counts during the whole follow-up period. The patients were recruited at different HIV-reference hospitals in Madrid, Spain. The recruitment period was 12 months. We started with the entire HIV population of each hospital, and the patients meeting the inclusion criteria and willing to participate in the study were selected. The study protocol was evaluated and approved by the Ethical review board of Instituto de Investigación Sanitaria-Fundación Jiménez Díaz, Madrid, Spain in accordance with the declaration of Helsinki. Each participating patient signed an informed consent form.

### Cell samples

We collected two 300 mL EDTA-anticoagulated blood samples (6 months apart) from each participant to purify the different memory CD4+ T cell populations (Trm, pTfh and non-pTfh cells). One sample was used to isolate Trm cells and the other sample was used to isolate pTfh and non-pTfh cells. All the analyses were conducted using fresh peripheral blood mononuclear cells (PBMCs) isolated by density gradient centrifugation with Ficoll-Hypaque (Sigma Chemical Co., USA).

### Immuno-magnetic purification of Trm, pTfh and non-pTfh cells

**Figure 3:**
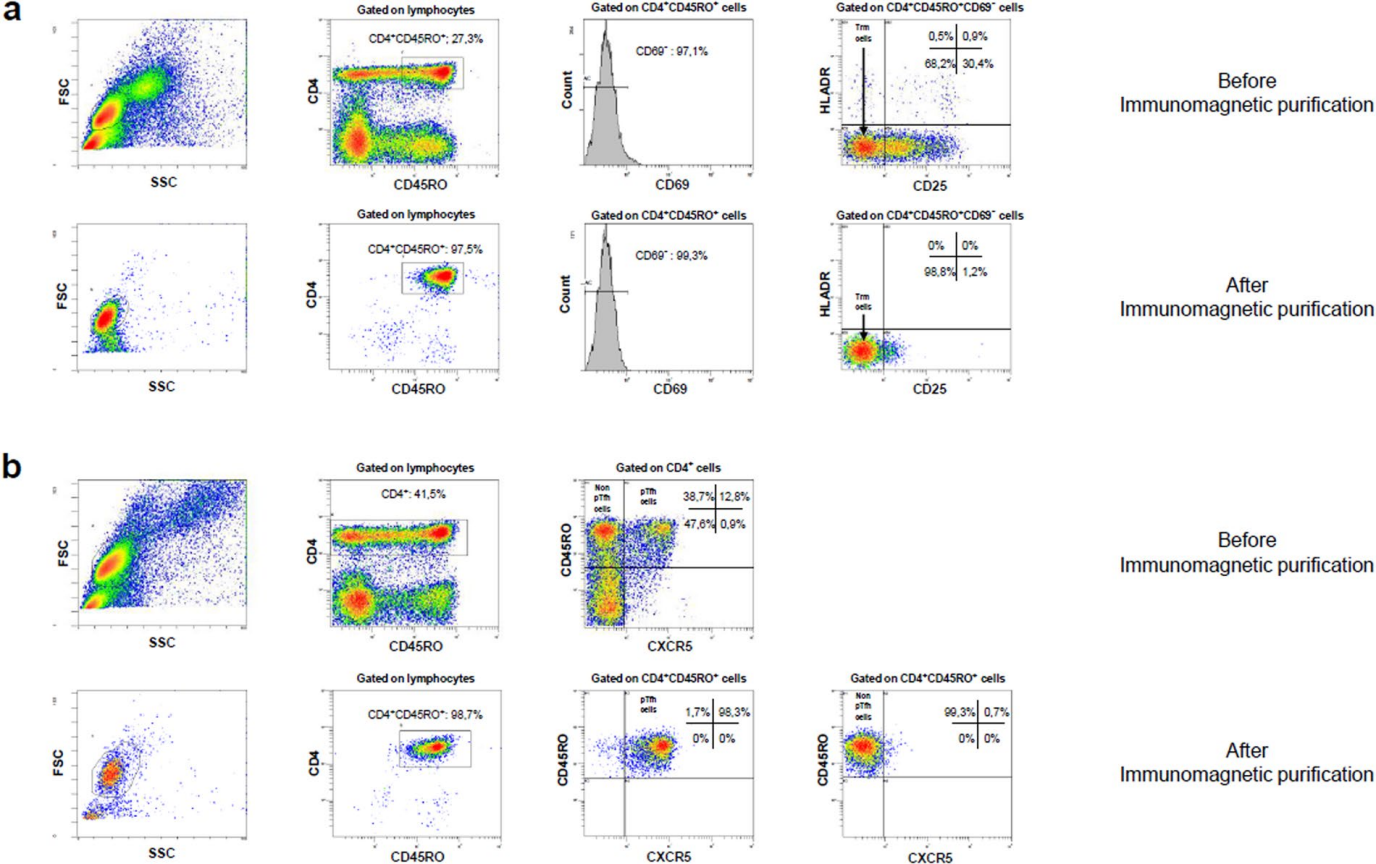
Representative flow cytometry example of immuno-magnetic purification of Trm cells (**a**) and of pTfh and non-pTfh cells (**b**) before and after purification. The gating strategy and percentages of different T-cell populations are shown. Trm cells were defined as CD4^+^CD45RO^+^CD69^−^CD25^−^HLADR^−^ cells. pTfh cells were defined as CD4^+^CD45RO^+^CXCR5^+^ cells and non-pTfh cells were defined as CD4^+^CD45RO^+^CXCR5^−^ cells.

We isolated the following different CD4+ T-cell populations from total fresh PBMCs (without any stimulation to avoid the triggering of TCR): Trm (defined as CD4+CD45RO+CD25−CD69−HLADR−), pTfh (defined as CD4+CD45RO+CXCR5+), and non-pTfh (defined as CD4+CD45RO+CXCR5−). The cells were isolated using an immuno-magnetic separation technique (MACS microbeads system, Miltenyi Biotec, Madrid, Spain). The purification of each one of these cell subsets was preceded by a negative magnetic isolation of memory CD4+ T-cell subset (memory CD4 T-cell Isolation Kit) according to the manufacturer’s instructions. Trm cells were isolated through negative selection with a cocktail of monoclonal antibodies against CD25, CD69, and HLA-DR. pTfh cells were purified through positive selection using monoclonal antibody against CXCR5 (Clon REA103, Miltenyi Biotec). The non-labeled cells were collected since they included the non-pTfh cells. The purity of each isolated CD4+ memory cell subset was assessed by flow cytometry (SP6800 Spectral Analyzer, Sony, USA) and the purity was greater than 90% in all samples. A flow cytometry experiment illustrating the gating strategies for Trm, pTfh and non-pTfh cell subsets, as well as the purity after isolation of each cell subset is shown in Fig. 3.

### Cell-associated HIV-DNA quantification

Total DNA from purified Trm, pTfh, and non-pTfh cells of each patient was extracted using QIAamp DNA-MiniKit (Qiagen, USA) following the manufacturer’s instructions. The size of the proviral DNA reservoir of each cell subpopulation was measured as intracellular HIV-1 DNA copies per million cells using ultrasensitive digital-droplet PCR (ddPCR) as previously described^44^. DdPCR is a technique that measures total HIV-DNA as a marker of the HIV reservoir^39,40^ and has greater precision and reproducibility in the context of a low level of detection (as is the case of EC patients) compared with techniques that measure integrated HIV-DNA^45^. The contributions of the pTfh and non-pTfh subsets to the HIV reservoir in total memory CD4+ T-cells were calculated by accounting for the level of HIV-DNA in each of these subsets and their proportion among total memory CD4+ T-cells.

### Statistical analysis

The main characteristics of the study groups are expressed as median [interquartile range]. Non-parametric Wilcoxon signed-rank test was used to evaluate differences between cell subpopulations among each group of patients, and Mann-Whitney U-test was used to evaluate the differences between patient groups in each cell subpopulation. All statistical analyses were performed using SPSS software version 15 (SPSS Inc., Chicago, IL, USA). All p-values were two-tailed and were considered significant only when lower than 0.05.

### Data availability statement

All data generated or analyzed during this study are included in this published article.
